# Impact of Fluid Flow Shear Stress on Osteoblast Differentiation and Cross-Talk with Articular Chondrocytes

**DOI:** 10.3390/ijms23169505

**Published:** 2022-08-22

**Authors:** Paige V. Hinton, Katelyn J. Genoud, James O. Early, Fergal J. O’Brien, Oran D. Kennedy

**Affiliations:** 1 Tissue Engineering Research Group, Department of Anatomy and Regenerative Medicine, RCSI University of Medicine and Health Sciences, D02 YN77 Dublin, Ireland; 2 Trinity Centre for Biomedical Engineering, Trinity College Dublin, D02 YN77 Dublin, Ireland; 3 Advanced Materials and Bioengineering Research (AMBER) Centre, D02 YN77 Dublin, Ireland

**Keywords:** osteoblast, chondrocyte, bone, fluid flow shear stress, cross-talk, post-traumatic osteoarthritis

## Abstract

Bone cells, in particular osteoblasts, are capable of communication with each other during bone growth and homeostasis. More recently it has become clear that they also communicate with other cell-types; including chondrocytes in articular cartilage. One way that this process is facilitated is by interstitial fluid movement within the pericellular and extracellular matrices. This stimulus is also an important mechanical signal in skeletal tissues, and is known to generate shear stresses at the micron-scale (known as fluid flow shear stresses (FFSS)). The primary aim of this study was to develop and characterize an in vitro bone–cartilage crosstalk system, to examine the effect of FFSS on these cell types. Specifically, we evaluated the response of osteoblasts and chondrocytes to FFSS and the effect of FFSS-induced soluble factors from the former, on the latter. This system will ultimately be used to help us understand the role of subchondral bone damage in articular cartilage degeneration. We also carried out a comparison of responses between cell lines and primary murine cells in this work. Our findings demonstrate that primary cells produce a more reliable and reproducible response to FFSS. Furthermore we found that at lower magnitudes , direct FFSS produces anabolic responses in both chondrocytes and osteoblasts, whereas higher levels produce more catabolic responses. Finally we show that exposure to osteoblast-derived factors in conditioned media experiments produced similarly catabolic changes in primary chondrocytes.

## 1. Introduction

Osteoblasts are highly specialized cells in bone tissue, which are derived from the mesenchymal lineage. Their primary function is to produce the collagenous substance known as osteoid, which subsequently becomes mineralized to form mature bone [1]. Osteoblasts are known to communicate with other cells, at various stages of differentiation within their own lineage (i.e., osteoprogenitors, bone-lining cells and osteocytes) [2,3,4,5]. However, more recently it has become clear that osteoblasts also communicate with other skeletal cell-types too; in particular articular chondrocytes in articular cartilage [5,6,7].

The concept of bone–cartilage crosstalk has gained significant attention in recent years, in particular in relation to joint injury and disease. However, the importance of this phenomenon is long established in the area of skeletal developmental biology. The role of osteoblasts as a crucial source of parathyroid hormone-related protein (PTHrp) during endochondral ossification, and their ability to communicate with hypertrophic chondrocytes in that process, is a central component of longitudinal bone growth [8,9]. In the mature adult joint, subchondral bone has also long been known to play a role in joint diseases like osteoarthritis (OA) and rheumatoid arthritis (RA). In those conditions, the subchondral region becomes either sclerotic or eroded depending on the stage (and type) of disease. However, in both those cases, these developments are most often late stage, or even end-stage symptoms, secondary to cartilage degeneration—and are thus seen to be of relatively little clinical or therapeutic utility. However, not all types of OA are equivalent in this regard. Post-traumatic OA (PTOA) occurs in the years following an acute joint injury (such as rupture of the anterior cruciate ligament (ACL)) and usually occurs in a relatively young cohort. In the immediate aftermath of these injuries (hours/days), subchondral bone damage in the form of bone marrow lesions (BMLs) result from the physical/mechanical injury [10]. BMLs can be visualized using specific MRI sequences and are reported in more than 80% of all acute knee injury cases. Their precise make-up and natural history have yet to be fully characterized. However, it is likely that they represent some kind of direct physical bone damage. Furthermore they have been linked with joint pain, and subsequent structural degradation of cartilage and other joint tissues [11]. Thus, it appears likely that direct mechanical damage to bone tissues [12,13], may indirectly affect cartilage tissue via bone–cartilage crosstalk.

One of the primary ways that bone cells perceive changes in their physical/mechanical environment is through fluid movement along their substrate/surface, and within the canaliculi/lacunar canalicular system (LCS) of the extracellular matrix (ECM). This allows for interstitial fluid flow shear stress (FFSS) to be generated which can change in magnitude in response to changes in the local micro-anatomy and topography [14]. This is a mechanism which gives rise to constantly changing shear stresses along the surfaces (targeting osteoblasts/lining cells), and throughout the LCS (targeting osteocytes)—making it an important mechanical stimulus in relation to osteoblast differentiation and activity. Specifically, osteoblasts at the bone surfaces are estimated to experience FFSS of around 0.8–1.0 Pa, while osteocytes within the LCS are estimated to experience shear stresses at a wider range of values between 0.8–3.0 Pa [15]. However, exact stress levels are difficult to determine due to the constantly changing micro-environment of the bone matrix [16]. Similarly, since water is the predominant constituent of articular cartilage, and cartilage is continuously loaded as part of everyday life, fluid movement and FFSS is also a central mechanical stimulus for cartilage cells [17]. Thus, the effect of mechanical stimulus on bone and cartilage cells, and the resultant cross-talk that occurs between them, is an important factor in understanding conditions like PTOA. However, this dual tissue system is also more complex to study in a controlled laboratory environment—thus novel methods and model systems will be important in attaining these goals.

Thus, the primary aim of this study was to develop and characterize an in vitro bone–cartilage crosstalk system, and its ability to respond to FFSS. Specifically, we evaluated the effect of oscillatory fluid flow on murine osteoblasts and chondrocytes via exposure to specific predetermined levels of FFSS, in terms of constitutive factor expression. We also studied the effect conditioned media from stimulated osteoblasts on chondrocytes. Our secondary aim was to assess the difference between cell lines and primary cells in this work [18,19]. We compared the responses of immortalized osteoblast and chondrocyte cell lines (MC3T3-E1 and ATDC5, respectively) to their primary counterparts when subjected to varying levels of FFSS. Finally, we demonstrated that soluble signals in conditioned media, from mechanically stimulated/damaged primary osteoblasts, can alter the phenotype of primary articular chondrocytes.

## 2. Results

### 2.1. Effects of Fluid Flow Shear Stress (FFSS) on the Proliferation and Differentiation of Primary and Cell Line Osteoblasts

**Figure 1:**
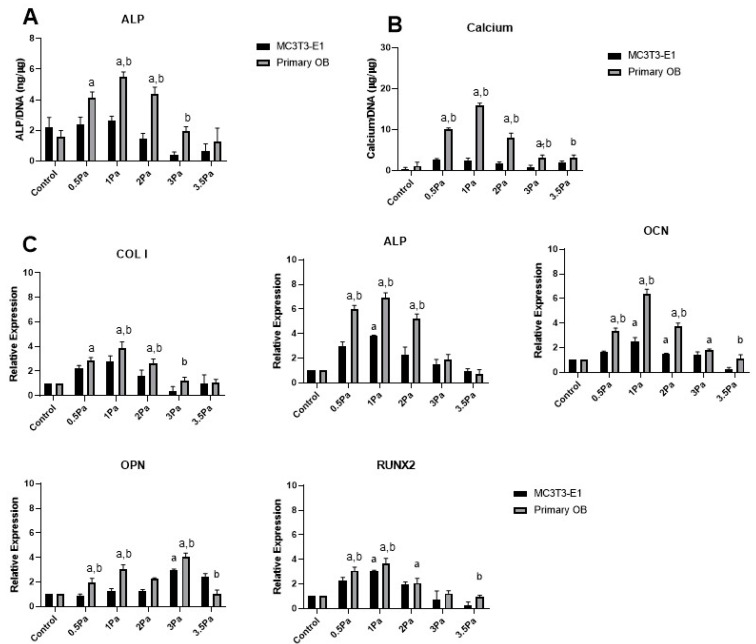
Effect of varying levels of FFSS on MSC-OBs and MC3T3s in terms of ALP, calcium and osteogenic gene expression. (**A**) ALP levels in both cell types at pre-determined FFSS levels, normalized to DNA content. (**B**) Calcium levels in both cell types at pre-determined FFSS levels, normalized to DNA content. (**C**) Gene expression levels for Collagen I, ALP, OCN, OPN and RUNX2 mRNA in MSC-OB cells via RT-PCR. Expression data were calculated using the 2^−ΔΔCt^ method relative to baseline levels and normalized using 18S as endogenous control. All regimes shown were carried out at a frequency of 1 Hz for a duration of 1 h. Statistical differences are shown where (a) represents significant difference compared with respective control/no-flow conditions, at a level of *p* < 0.05 and (b) represents significant difference compared with cell-line counterpart under identical FFSS conditions, at the level of *p* < 0.05.

Oscillatory FFSS in the 0.5–3.5 Pa range was applied to both cell line and primary osteoblast populations (MC3T3 and MSC-OB, respectively). Both cell types showed a marked increase in ALP and calcium immediately following stimulation up to FFSS levels of 1Pa (Figure 1A,B). However, at stress levels greater than 1 Pa, ALP and calcium both declined progressively. Furthermore, ALP and calcium levels were significantly greater in the MSC-OB group compared with MC3T3s in the majority of cases, showing that primary cells respond to this stimulus in a more robust manner than their cell line counterparts.

Data in Figure 1A,B show a remarkably consistent trend in ALP and calcium levels. Furthermore, the peak level for both factors, in primary cells, was at 1 Pa—which is precisely the magnitude of FFSS to which they are exposed in situ [15]. Similarly, gene expression data showed that the peak level, for all osteogenic factors examined (except OPN), was also at 1 Pa (Figure 1C). Once again it was also noted that in the majority of cases, the magnitude of the response was greater in primary cells, compared with cell line counterparts. Moreover, most genes that were measured showed significantly reduced expression at higher levels of FFSS (3–3.5 Pa) with the exception of OPN which was found to increase marginally at this level. While OPN is an important marker of bone formation some studies have identified it as particularly sensitive to mechanical loading [20], which could explain this response. Overall, these data indicate that low levels of shear stress produce a stimulatory, or an osteogenic, response up to 1 Pa. However, beyond that threshold, those effects are reversed as a function of increasing FFSS. This can be viewed as a ‘damaging’ effect to which these cells respond in a negative way. Thus, for simplicity and clarity, we refer to the 1 and 3 Pa regimes as ‘stimulatory’, and ‘damaging’, respectively.

**Figure 2:**
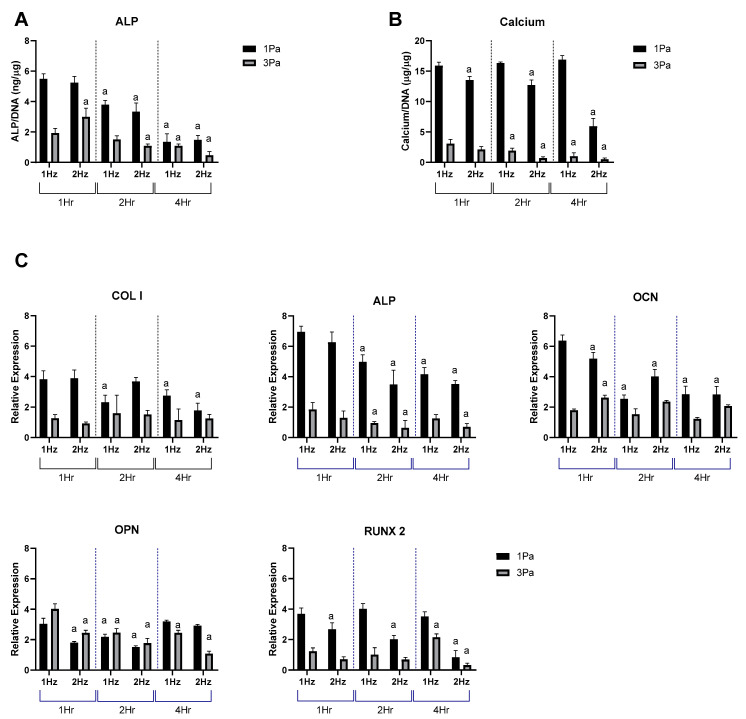
Effect of frequency and duration of FFSS conditions on MSC-OBs at 1 and 3 Pa in terms of ALP, calcium and osteogenic gene expression. (**A**) ALP levels normalized to DNA content. (**B**) Calcium levels normalized to DNA content. (**C**) Gene expression levels for Collagen I, ALP, OCN, OPN and RUNX2 mRNA in MSC-OBs via RT-PCR. Expression data were calculated using the 2^−ΔΔCt^ method relative to baseline levels and normalized using 18S as endogenous control. Statistical differences are shown where (a) represents significant difference compared with conditions at the same stress level at 1 Hz and 1 h, to the level of *p* < 0.05.

Based on the data shown in Figure 1, we carried out a study to determine and optimize the effect of frequency and duration of the oscillatory flow parameters on the stimulatory (1 Pa) and damaging (3 Pa) regimes in primary cells (Figure 2). The FFSS used in the stimulatory regime produced an anabolic response, whereas the damaging regime resulted in a more catabolic response profile, with increases in markers of de-differentiation and hypertrophy. With change of frequency and duration it was shown that, as expected, there was a significant difference between 1 and 3 Pa at each of the frequencies examined (1 and 2 Hz) and at each time points (1, 2 and 4 h). It was also noted that there was a reduction in expression levels at 2 Hz compared to 1 Hz, at the majority of time points—particularly at longer durations. It was also noted that, calcium content and Col I, RUNX2 and OPN were relatively stable over time for each cell type.

### 2.2. Effects of FFSS on the Proliferation and Differentiation of Chondrocytes

**Figure 3:**
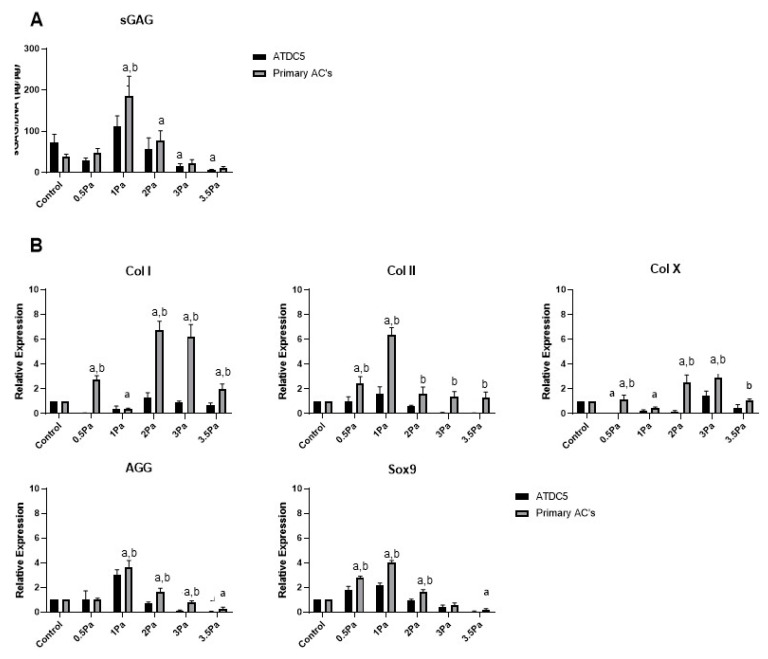
Effect of varying levels of FFSS on primary ACs and ATDC5 cells. (**A**) sGAG levels of both cell types normalized to DNA content. (**B**) Gene expression levels for collagen type I, II, and X, SOX9, and aggrecan mRNA via RT-PCR. Expression data were calculated using the 2^−ΔΔCt^ method relative to baseline levels and normalized using 18S as endogenous control. All regimes were carried out at a frequency of 1 Hz for a duration of 1 h. Statistical differences are shown where (a) represents significant difference compared with respective control/no-flow conditions, at a level of *p* < 0.05 and (b) represents significant difference compared with cell-line counterpart under identical FFSS conditions, at the level of *p* < 0.05.

Distinct changes were also observed in the response of primary Articular Chondrocytes (ACs) to FFSS, compared with no-flow controls (Figure 3). Cells were exposed to FFSS regimes (0–3.5 Pa) in a similar way to the previous experiments. Initially, there was no significant differences between AC and ATDC5 cells in their sGAG content. However, as before 1 Pa seemed to represent a threshold level, where this parameter peaked in primary chondrocytes. Also as before, at all higher FFSS magnitudes, sGAG levels were progressively reduced. Gene expression data showed that Col II, AGG and Sox9 also reached a peak at 1 Pa—this time with clear differences between cell types. In contrast, Col I and Col X reached their peaks at 2/3 Pa, respectively. In all cases the primary chondrocytes displayed a more robust response compared to that of their cell line counterparts.

**Figure 4:**
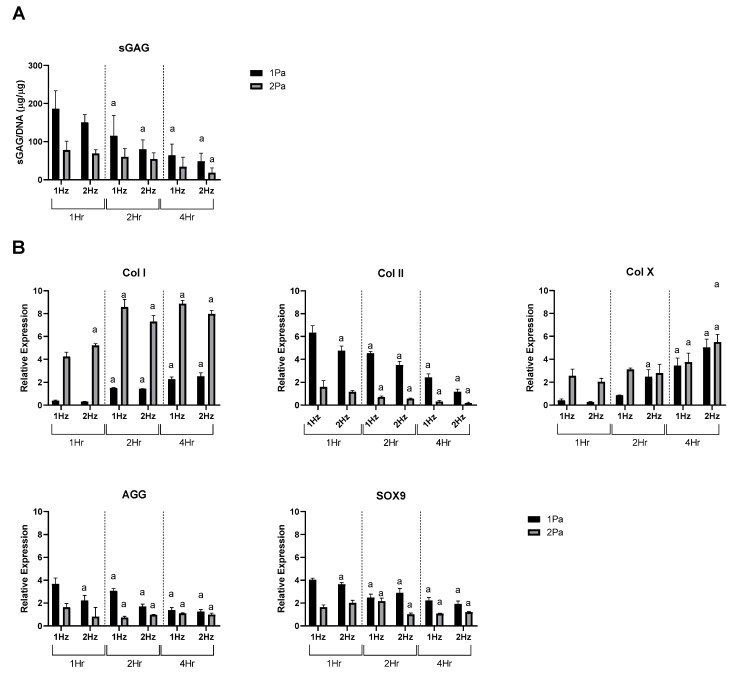
Effect of frequency and duration of FFSS conditions on primary ACs at 1 and 2 Pa on sGAG and gene expression. (**A**) AC sGAG levels normalized to DNA content. (**B**) Gene expression levels for collagen type I, II, and X, SOX9, by ACs via RT-PCR. Expression data were calculated using the 2^−ΔΔCt^ method relative to averaged baseline samples and normalized using 18S as an endogenous control. Statistical differences are shown where (a) represents a significant difference compared with conditions (1 or 2 Pa) at 1 Hz at 1 h, to the level of *p* < 0.05.

Based on the data presented in Figure 3 the stimulatory (1 Pa) and damaging (2 Pa) models were then used to determine how primary ACs responded to change of frequency and duration as before (Figure 4). We found that increasing frequency and duration tended to decreased sGAG content and Col II, AGG and SOX9 expression at each stress level while conversely increasing hypertrophic markers Col X and Col I under the same conditions.

### 2.3. Effect of FFSS Induced Osteoblast Derived Soluble Factors on Chondrocytes

**Figure 5:**
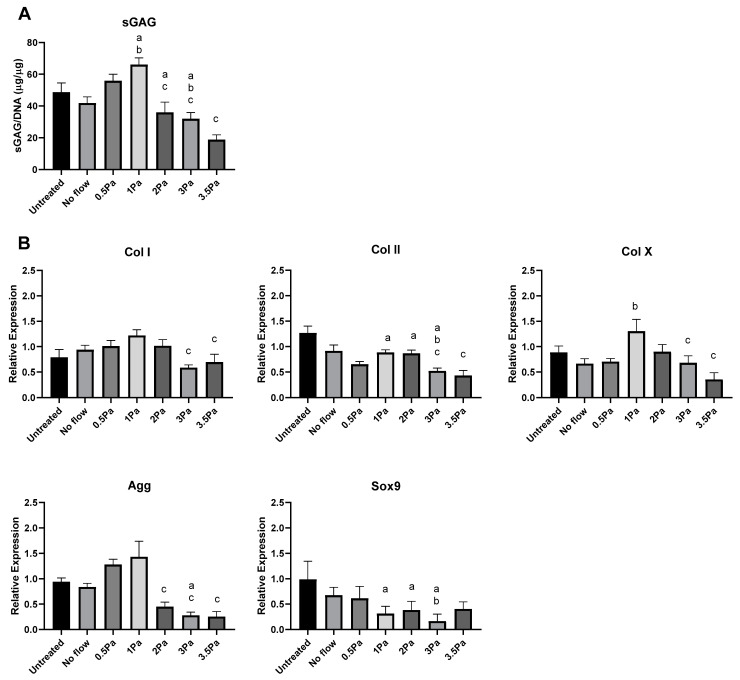
Changes in chondrocyte phenotype due FFSS-induced soluble osteoblast-derived factors. (**A**) sGAG levels in ACs following exposure to FFSS-induced soluble osteoblast-derived factors, normalized to DNA content. (**B**) AC gene expression of collagen I, II, and X, SOX9, and aggrecan at 3 day following exposure to conditioned media. Expression data were calculated using the 2^−ΔΔCt^ method relative to averaged day 1 samples and normalized using 18S as endogenous control. All regimes were at 1 Hz for 1 h. Statistical differences are shown where (a) represents significant difference compared with untreated control (b) represents significant difference compared with no-flow control and (c) represents significant difference compared with 1 Pa, to the level of *p* < 0.05.

Finally, we sought to determine the effect of FFSS-induced osteoblast-derived soluble factors on chondrocytes, using conditioned media experiments. We first generated conditioned media from MSC-OBs exposed to the same spectrum of FFSS magnitudes as in previous experiments. That conditioned media was then collected and applied to healthy ACs, and we carried out biochemical and gene expression analyses as before to assess the impact of soluble osteoblast-derived factors on chondrocyte phenotype. The data in Figure 5 show that at stimulatory FFSS levels (i.e., 1 Pa), soluble osteoblast-derived factors resulted in increased sGAG production in chondrocytes compared to control levels. However, soluble factors generated in response to FFSS at 2, 3 and 3.5 Pa caused sGAG content to be reduced compared to control and peak levels, and this pattern was also found for aggrecan at the gene level. Surprisingly, the increase in Col II gene expression at 1 Pa was not evident in this system, however, a reduced expression compared to it, and controls was observed at 2, 3 and 3.5 Pa, as before. Similarly, expression of SOX9 was also reduced compared to controls at high FFSS levels. The response of Col I showed a non-significant increase in expression up to 2 Pa while at 3 and 3.5 Pa expression was reduced compared to 1 Pa. In the case of Col X, the expression profile was a somewhat similar to Col I with a peak at 1 Pa followed by significant reductions at 3 and 3.5 Pa.

These data show that exposure to stimulated osteoblast-derived factors can have a significant magnitude-dependent effect on AC phenotype.

## 3. Discussion

The mechanisms through which skeletal cells, such as osteoblasts, respond to mechanical stimuli can affect the health of bone and cartilage tissues individually, but also whole joints and their response to injury and disease. Addressing this question can be complex due the interactions between different cell types, and also the influence of their respective local microenvironments. The overall aims of this study were to determine the effect of Fluid Flow Shear Stress (FFSS) on osteoblasts and chondrocytes, and also to determine the effect of soluble factors produced by stimulated osteoblasts on chondrocytes via conditioned media experiments. Initially we compared primary osteoblasts and chondrocytes, to their cell-line counterparts (MC3T3-E1 and ATDC5, respectively) to determine the optimal cell type for our application. Thus, we established an in vitro system to study the effect of FFSS on bone and cartilage cells and also a system to study the interaction between the two.

Applying external forces to cells in vitro introduces additional complexity to systems that are already sensitive to their environment. Then, establishing cell–cell communications can introduce yet more complexity and variability, especially when using cell-lines. Thus, it was a critical step to isolate a consistently pure population of primary osteoblasts and articular chondrocytes to ensure consistency in these studies. For osteoblasts, a number of isolation methods were explored (data not shown), and we found that a bone marrow-derived MSC isolation and differentiation protocol produced the most consistent data [21]. This approach produced reproducible cell yield and a stable cell population with reliable osteogenic potential with strong differentiation over a 28-day period and increased calcium levels as well as osteocalcin production—which we found to be a reliable constitutive osteoblast marker [22]. Once fully differentiated, MSC-OBs cells produce and co-ordinate mineralization of the bone matrix.

Few direct comparisons have been reported between MC3T3 cells and their species-specific primary cell counterpart. However some key studies have identified significant reductions in constitutive OB markers in MC3T3 cells compared to primary cells (human osteoblasts [23,24]). These studies highlighted differences in proliferation, ALP, calcium, and key phenotypic markers, such as Col I, OCN and RUNX2. Though proliferation of cell lines is typically considered superior, stress responses to extrinsic stimuli is often diminished, thus the requirement to rigorously test this aspect against primary cells in this study.

Therefore, we required cell populations that could maintain a stable production of these key osteogenic factors for duration of our experiments. ALP activity is an early marker of osteogenic differentiation and plays a role in mineralization via enzymatic hydrolysis activity [25,26]. Our data showed a significant increase in ALP in response to osteogenic factors, when compared with the MC3T3 cells. We also found they produced increased levels of ALP over a 28-day period compared with the cell line. A further indication of advancing maturity in MSC-OBs was their capacity for increased production of calcium compared to MC3T3s. We found this parameter followed a similar trend to ALP in differentiating MSCs with a significant increase in calcium following the addition of osteogenic factors. When compared with MC3T3 cells, we found these cells produced lower levels of calcium initially, but reached comparable level over the 28-day period—suggesting a compensatory mechanism at later time points.

Our gene expression data showed an increase in Col I, ALP, OPN, OCN and RUNX2 in our primary population compared with the cell line. These data were in line with our expectations and compare well with other data reported in the literature, whereby primary cells produce increased levels of these factors and maintain their native physiological responses [21], albeit with these responses diminishing over time. These data show that we have developed a reliable and reproducible method for producing pure MSC-derived OBs that reliably differentiate into healthy mechanoresponsive populations. In comparison to immortalized MC3T3s, our primary populations demonstrated increased proliferation and increased production of cellular ALP and calcium.

Consistent populations of articular chondrocytes can also be difficult to isolate, culture and maintain [27]. This often manifests in culture with a strong tendency towards dedifferentiation and a fibroblastic state. Once this occurs, fibroblastic cells overtake the culture—making data from any subsequent studies difficult to interpret. Our ACs were isolated from the fore- and hind-limbs of C57Bl6 mice. We also initially tested an isolation protocol from costal cartilages [28,29] and compared both methods (data not shown) but determined that appendicular ACs were superior. We optimized our system for pure AC cell populations using morphological, biochemical, immunofluorescence, and gene expression analyses. Those data sets showed that we have established a robust system for AC isolation with morphological data showing cuboidal/mosaic shaped cells consistent with articular chondrocytes and histochemical staining showing representative nuclei, collagen fibrils, and acidophilic cytoplasm. While markers for fibroblastic/hypertrophic dedifferentiation increased after certain timepoints, it was clearly possible to avoid that scenario by limiting the duration of experiments. Immunocytochemistry assays also demonstrated strong positive staining for Col II, which is constitutively expressed by healthy ACs.

Since the development of immortalized cell-lines, many musculoskeletal cell types have been developed. Although these were a crucial development for certain aspects of cell biology research it is now widely accepted that they can be substantially different from their primary cell counterparts in key ways. For example, when comparing against the ATDC5 cell line, our primary cells showed a significantly different protein and gene expression profile. Our primary cells were more representative of the physiological in situ conditions which we were trying to recapitulate. However, as described above, these primary cells can be difficult to isolate and maintain in a monolayer due to their tendency to dedifferentiate in response to stress, and they also undergo senescence processes relatively quickly and can have limited abilities for self-renewal. We found that these cells develop morphological, gene expression and functional changes as a function of age. This was reflected in our gene expression studies where initially high levels of collagen type II and aggrecan, decreased significantly over a 28-day period. These data compare favorably to similar studies in the literature and confirm that our AC isolation procedure yields pure articular chondrocytes, despite passaging over time [30,31]. Our data also demonstrated that while MC3T3 and ATDC5 cells are useful for recapitulating, and studying, osteoblast and chondrocyte behavior, respectively—they exhibit a significantly different gene expression profile to primary cells and their responses to important mechanical stimuli are also different, thus this should be considered at the experimental design stage of studies using these cells.

Musculoskeletal tissues are well known to be highly mechanosensitive, and shear stress in particularly can often have a particularly potent effect on osteochondral tissues and cells. Pre-clinical studies have demonstrated that both bone and cartilage require consistent levels of mechanical stimulation to maintain normal functionality (such as BMU mediated remodeling in bone, and collagen turnover in cartilage). However, overloading can cause injury and disease, although the precise threshold between these is often challenging to define. The experimental use of FFSS has been particularly important in addressing these issues, since it can be used to deliver controlled shear stresses, similar to those experienced at the cellular level, to a high degree of accuracy. Broadly speaking it has been shown that lower stress levels result in stimulatory responses in osteochondral tissues while higher stresses result in damaging or negative responses. This seems intuitively correct based on our existing knowledge of skeletal mechanobiology and is also in accordance with our data in this study. Our MSC-OB cell populations responded to oscillatory FFSS with rapidly increased production of ALP, calcium, OCN and RUNX2. These data are consistent with data in the literature suggesting that shear stress at low levels (<1 Pa) increases OB proliferation and osteogenic differentiation [32,33,34,35]. These results indicate lower shear stress levels have a generally positive stimulatory effect on this population, but as discussed above, a more pronounced increase in the expression level was observed in the primary population compared to the cell line (Figure 1). In contrast, at higher shear stress levels a reduction of pro-osteogenic markers was observed, with decreases in ALP, calcium, Col I, OCN and RUNX2 expression. Taken together, these data suggest this regime represents a more damaging profile for these cells than the 1 Pa group—this again compares well with the existing literature [19,36,37,38] . We also found that maintaining FFSS at 1 Hz for 1 h generated a stimulatory regime of 1 Pa but a damaging regime at 3 Pa. We used these parameters to determine if altering frequency and duration had an effect on the MSC-OBs (Figure 2). It was observed that 2 Hz regimes had a lower expression level compared to the 1 Hz group. We also noted a general decrease in expression levels with increased duration, however this was not as marked as the frequency changes. To our knowledge, this effect has not been previously reported in MSC-OBs under these conditions. Although, a similar study by Li et al. 2012 showed similar effects as a result of modulation of frequency and duration of flow parameters on osteocytes [39].

FFSS is also an extremely relevant stimulus for chondrocytes, although this cell type likely has different basal levels and response rates to stimulus. Previous studies have shown that mechanical stimulation can be employed to improve matrix composition and production in cartilage tissue engineering and as such is considered an effective and important functional strategy for regenerative medicine [40,41]. Similar to our osteoblast studies, our data show that primary chondrocytes exposed to varied levels of shear stresses respond more robustly than their cell line counterparts. This is also in accordance with the literature and suggests once again that primary cells are more responsive and sensitive to important changes in physiologically relevant shear stress levels [27]. A number of studies have shown that low FFSS levels support cell growth and tissue regeneration [42,43,44,45,46], for example a perfusion bioreactor was used to generate shear stresses on tissue constructs which showed increased cartilage tissue production with matrix deposition and cell proliferation [43,44]. Elsewhere, when a bioreactor was used for similar purposes, increased cartilage development primarily near the surfaces exposed to shear stress was observed after as little as 8 days, with collagen deposition and proteoglycan deposition being used as a proxy for cartilage development [45].

Although we used a different system for the application of FFSS, our data also showed an increase in chondrogenic factors (Col II and aggrecan) in response to this stimulus, in line with these previous studies. This supports the idea that tissue response is not dependent on the method of shear stress generation but is specifically to its magnitude and duration (Figure 3 and Figure 4). We also found that under conditions of high FFSS, gene expression profiles changed fundamentally, with increases in Col I and X expression, reduction Col II expression and decreased GAG content (Figure 3 and Figure 4). This can be described as a damaging regime, or at least one that induces a de-differentiation or hypertrophic pressure on these cells. These data are also consistent with the literature, for example Zhu et al. determined that high FFSS recapitulates chondrocyte gene expression profiles to those associated with pathological conditions such as OA [47]. This supports the idea that increased shear stresses, induced via FFSS is an important factor in OA development.

In the more specific context of PTOA, it is currently assumed that at the time of acute joint injury (e.g., ACL rupture), there is little direct (mechanical) damage to cartilage/chondrocytes. This tissue is designed and optimized to withstand and dissipate impact forces such as these. However, what is often observed as a direct result of joint injury, and in its immediate aftermath, is mechanical damage to the subchondral bone in the form of BMLs. These form as a direct result of injury and have been linked with pain and structural joint changes [10,11,12,13]. Later during disease development, abnormal bone remodeling modulates the local microenvironment further, but in a different way. Nonetheless both these responses could potentially alter the local interstitial fluid flow in the region, which may then play a role in indirectly affecting cartilage tissue health via bone–cartilage crosstalk. Previous studies have shown that under certain conditions, specific factors are capable of crossing the osteochondral interface and the level and extent of crosstalk could be increased as a function of damage within the joint [48,49,50]. Overall, our gene expression studies showed similar trends to those reported previously, where anabolic gene expression decreases with increasing FFSS. Further to this, our study shows that MSC-OBs, stimulated at varying FFSS levels, produce soluble factors that can modulate chondrogenic factor production in healthy articular chondrocytes, with a transition point (between anabolic and catabolic phenotypes) occurring at around 1 Pa (Figure 5). This study has some limitations which are important to consider. For example, in the case of Col II expression (which is the standard marker for healthy chondrocytes), we found limited increase in its expression in conditioned media experiments even though a reduction from 1 Pa was observed (i.e., at 2, 3 and 3.5 Pa). Similarly, the Col X expression profile was unexpected in that the maximum value came at 1 Pa in this study. This is difficult to interpret fully, however does indicate some cells being directed down a hypertrophic line under these conditions.

In the future, it will be important to identify and more broadly understand the constituents of the secretome/mechanosome, and in particular those individual elements responsible for the phenotypic changes seen in chondrocytes in response to FFSS-induced bone-derived factors. It will also be important to address other categories of factors which are likely to be involved in bone–cartilage crosstalk, such the paracrine factors nitric oxide (NO), PGE2 and COX-2. With the identification of these factors, and their roles, it may be possible to generate new targets and to prevent deterioration of chondrocytes despite damage to subchondral bone. This approach could help prevent PTOA development post-acute joint injury.

## 4. Materials and Methods

### 4.1. Cell Culture

#### 4.1.1. Cell Line Culture

MC3T3-E1 cells were obtained from American Type Culture Collection (ATCC, Manassas, VA, USA) and maintained in Minimum Essential Medium Eagle—Alpha Modification (α-MEM) supplemented with 2 mM L-glutamine, 100 U/mL penicillin, 100 μg/mL streptomycin, and 10% FBS. Ascorbic acid (50 μg/mL), β-glycerophosphate (10 mM) and dexamethasone (100 nM) (Sigma-Aldrich, Arklow, Ireland) was added for osteogenic media. A sub-cultivation ratio of 1:6 to 1:8 was preformed every 3–4 days.

ATDC5 cells, derived from mouse teratocarcinoma AT805 cells (Sigma-Aldrich, Arklow, Ireland)were maintained in DMEM: Ham’s F12 (1:1), 2 mM Glutamine, 1× ITS (10 μg/mL recombinant human insulin, 5.5 μg/mL human transferrin (substantially iron-free), and 5 × 10^−3^ μg/mL sodium selenite) (Sigma-Aldrich, Ireland), 100 U/mL penicillin, 100 μg/mL streptomycin, and 5% FBS. A sub-cultivation ratio of 1:6 was preformed every 3–4 days.

#### 4.1.2. Primary Osteoblast Isolation

To generate primary osteoblasts (MSC-OB) hind-limbs were isolated from 8 week adult C57Bl/6 mice and the joints were disarticulated, according to an established protocol [21]. Individual bones were separated, cleaned, and the epiphyses removed. Marrow cavities were flushed with 10 mL of media. Samples were centrifuged and the pellet was resuspended in 3 mL red cell lysis buffer (Sigma-Aldrich, Arklow, Ireland) for 5 min. Culture media was added to neutralize the lysis buffer and centrifuged. These cells were cultured in DMEM high glucose, supplemented with 2 mM L-glutamine, 100 U/mL penicillin, 100 μg/mL streptomycin, and 10% FBS. After 7 days, the cells were cultured in osteogenic media α-MEM supplemented with 2 mM L-glutamine, 100 U/mL penicillin and 100 μg/mL streptomycin, 10% FBS, ascorbic acid (50 μg/mL), β-glycerophosphate (10 mM) and dexamethasone (100 nM) (Sigma-Aldrich, Arklow, Ireland).

#### 4.1.3. Primary Chondrocyte Isolation

The hind limbs of P5 C57Bl/6 mice were isolated and the cartilaginous humeral, femoral heads, condyles and tibial plateaus were removed. Chondrocytes harvested from these areas are termed Articular Chondrocytes (ACs). Tissues were digested in a 3 mg mL^−1^ collagenase digestion (Dulbecco’s Modified Eagle’s Medium (D5546) supplemented with 2 mM L-glutamine, 50 U/mL penicillin and 0.05 mg/mL streptomycin), repeated, and then reduced to 0.5 mg mL^−1^ overnight. Cells were cultured using chondrocyte cell media supplemented with FBS, L-glut and penicillin-streptomycin.

### 4.2. In Vitro Fluid Flow Shear Stress Model

A commercial IBIDI^®^ fluid flow system (Ibidi GmbH, Munich, Gräfelfing, Germany) was used to deliver a controlled level of fluid flow shear stress to bone/cartilage cells. The system consists of a syringe pump (a positive/negative displacement system), connected to an IBIDI µ-Slide VI0.4 and syringe media reservoirs at inlet and outlets. Sterile media was delivered into slides to prime the system for each experiment. Cells were added at a density of 1.5 × 10^3^ cells/mL 5 days prior to flow experiments. Once the circuit was closed and confirmed, cells in the chambers were exposed to FFSS at pre-determined flow rates and duration (Table 1). Parameters were set to ensure accurate testing, where by >50% of adhered cells under pre-test conditions must be present post-test [39].

The magnitudes of FFSS were selected to represent a range covering stimulatory regimes and damaging regimes depending on cell type.

#### 4.2.1. Preparation of Conditioned Medium (CM) from Osteoblasts

Mature MSC-OBs were seeded into IBIDI µ-Slide VI0.4 channels at a density of 1.5 × 10^3^ cells/mL and left to attach overnight. Cells were cultured in growth medium for 3 days to allow the cells to proliferate and reach 80% confluence. In order to prepare conditioned medium, cells were washed twice in PBS and incubated for another 24 h in growth medium. The media was harvested after 24 h, centrifuged at 1200 rpm for 5 min and passed through a 0.2 µm filter to remove any remaining cell debris. The media was transferred to endotoxin-free Eppendorf’s and stored at −80 °C until further use. Next, mature MSC-OBs were seeded into IBIDI µ-Slide VI0.4 channels at a density of 1.5 × 10^3^ cells/mL and left to attach overnight. Cells were cultured in growth medium for 3 days to allow the cells to proliferate and reach 80% confluence. Once confluence was reached, cells were subjected to FFSS of 0.5, 1, 2, 3 or 3.5 Pa. our system allowed 2 channels to be flowed at a time with oscillatory flow (LEGATO^®^ 210P syringe pump, KD Scientific, Holliston, MA, USA). After set regime was completed, cells were incubated for 24 h to allow cells to equilibrate post-flow. The media was harvested, centrifuged at 1200 rpm for 5 min and passed through a 0.2 µm filter to remove any remaining cell debris. The media was transferred to endotoxin-free Eppendorf’s and stored at −80 °C until further use.

#### 4.2.2. Experimental Set-Up for Conditioned Media Studies

**Figure 6:**
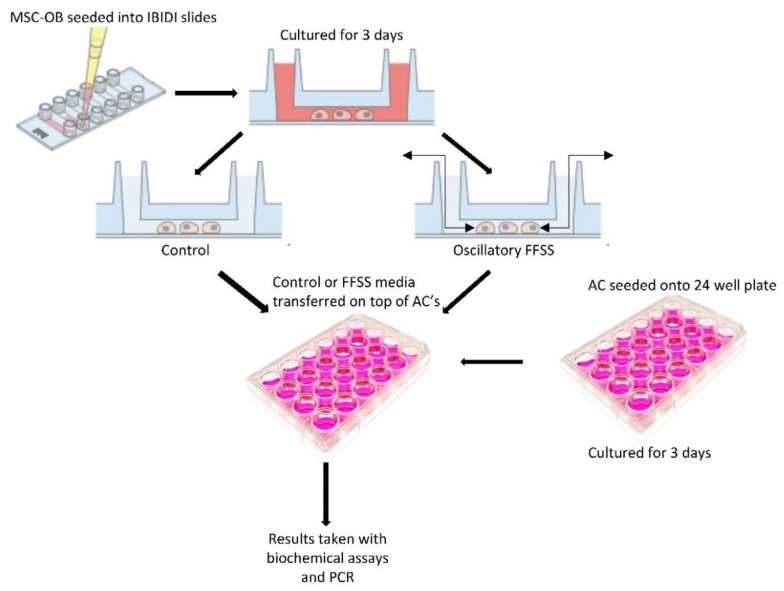
Schematic of conditioned media experiments. Cells were seeded into IBIDI slides, cultured for 3 days prior to FFSS or sedentary control before conditioned media was collected and introduced. Adapted with permission from [51].

ACs were seeded on 24 culture-treated well plates at a density of 30,000 cells/cm^3^ and maintained as described above. Following overnight incubation, media was changed, and cells cultured for a further for 3 days to allow proliferation to 80% confluence. Cells were washed with PBS twice and incubated with either untreated control, conditioned media control or conditioned FFSS media for 24 h (Figure 6). The media and cells were harvested for sGAG and Pico green analysis as previously below. The cells were further analyzed via qPCR for gene analysis below.

### 4.3. Biochemical Assays

#### 4.3.1. Biochemical Analysis of Osteoblast Cells

Cells were thawed, homogenized in 1 mL of lysis buffer consisting of PBS supplemented with 2% Triton-x-100 and centrifuged for 15 min at 10,000× *g* and 4 °C. DNA quantification was carried out using a Quant-iT™ PicoGreen^®^ dsDNA assay kit (Biosciences, Dublin, Ireland) with a Lambda DNA standard, at excitation at 485 nm and absorption at 538 nm was used. Alkaline phosphatase activity was measured using a Sensolyte pNPP Alkaline Phosphatase assay kit (Cambridge Bioscience, Cambridge, UK) against a calf intestine alkaline phosphatase standard. Enzymatic activity was measured for absorbance at 405 nm and normalized against standard controls [52]. Calcium content was measured by digesting samples in 1 mL of 0.5 M hydrochloric acid (HCL) and using a StanBio Calcium Liquicolour Kit (ThermoFisher Scientific, Waltham, MA, USA) according to the manufacturer’s instructions [53]. Absorbance was read at 595 nm.

#### 4.3.2. Biochemical Analysis of Chondrocyte Cells

For the Quant-iT™ PicoGreen^®^ dsDNA assay on chondrocytes, DNA standards and sample dilutions were prepared according to manufacturer guidelines; 100 μL of the appropriate standard or sample was pipetted into a black 96 well plate in duplicate and 100 μL of PicoGreen^®^ solution was then added to each well, and the plate was incubated at RT for 2–3 min. The plate was then read on the spectrofluorophotometer plate reader at the appropriate wavelength. Next, the Blyscan Assay (a quantitative dye-binding method to measure sulfated proteoglycans and glycosaminoglycans, (sGAG)) was used [28]. Cells were digested with papain lysis buffer and the assay was completed as per manufacturer’s instructions.

### 4.4. qPCR

RNA was isolated using an RNeasy Minikit (Qiagen, Hilden, Germany). Reverse transcription was performed with QuantiTect Reverse Transcription kit (Qiagen, Germany) followed by plating 2 μL cDNA and SYBR Green Master Mix (Qiagen, Germany) with appropriate primers in 96-well plates. The relative expression levels of a suite of candidate genes (Table 2) were calculated based on the standard 2^−∆∆**Ct**^ method.

### 4.5. Statistics

All statistical analyses were preformed using GraphPad V8.0.1 (GraphPad Software, Inc., San Diego, CA, USA). A one-way analysis of variance (ANOVA) was preformed when considering one cell type per analysis (Tukey’s post-hoc test) and two-way ANOVA was performed with a Sidak’s post-hoc test when comparing two different cell populations on different regimes or time points. Data were presented as mean values ± the standard deviation of the mean of 3 independent samples unless otherwise stated. *p* < 0.05 was considered significant.

## 5. Conclusions

In conclusion, we have developed a novel primary cell-based system that can be used to recapitulate osteoblast damage/stress after joint injury, and the subsequent deleterious effects this may have on chondrocytes via bone–cartilage crosstalk. In future, the specific pathways involved in this process can be used to determine bone-derived factors that produce a catabolic affect in cartilage cells. This information could also inform novel bone targeting biologic treatment for PTOA and other related diseases.

## Figures and Tables

**Table 1:** Shear Stress table for μ-Slide VI 0.4 for viscosity η = 0.0072 dynes/cm^2^ = 0.00072 Pa.

τ [Pa]	Φ [mL/min]	Displacement [µL]
0.5	3.94	63
1	7.89	127
2	15.77	253
3	23.66	380
3.5	27.60	447

**Table 2:** List of primer names, oligo sequences and accession numbers.

Primer Name	Forward Sequence	Reverse Sequence	Accession Number
Mouse Collagen I	5′ CTTCACCTACAGCACCCTTGTG 3′	5′ GATGACTGTCTTGCCCCAAGTT 3′	NM_007742.4
Mouse Collagen II	5′AAGTCACTGAACAACCGATTGAGA 3′	5′ AATGCGAGCAGGGTTCTTG 3′	NM_031163.4
Mouse Collagen X	5′ TTCTCCTACCACGTGCATGTG 3′	5′ AGGCGTGCCGTTCTTATACAG 3′	BC_15156930.1
Mouse Aggrecan	5′ GCCACGGTGCCCTTTTTAC 3′	5′ GAGAGAGGCGAATGGAACGA 3′	NM_007424.34
Mouse SOX9	5′ GGTGGAGTAGAGCCCTGAGC 3′	5′ CCTTCAACCTTCCTCACTACAGC 3′	NM_011448.4
Mouse OCN	5′ AAGCAGGAGGGCAATAAGGT 3′	5 ′ TTTGTAGGCGGTCTTCAAGC 3′	L24429.1
Mouse OPN	5′ AGCAAGAAACTCTTCCAAGCAA 3′	5′ GTGAGATTCGTCAGATTCATCCG 3′	J04806.1
Mouse OPN	5′ AGCAAGAAACTCTTCCAAGCAA 3′	5′ GTGAGATTCGTCAGATTCATCCG 3′	NM_0012040201.1
Mouse RUNX2	5′ ACTCTTCTGGAG CCGTTTATG 3′	5′ GTGAATCTGGCC ATGTTTGTG 3′	NM_001146038.2
Mouse ALP	5′ AACCCAGACAC AAGCATTCC 3′	5′ GAGAGCGAAGGG TCAGTCAG 3′	NM_007431.4
Mouse BMP2	5′ ACACAGCTGGTCACAGATAAG 3′	5′ CTTCCGCTGTTTGTGTTTGG 3′	L25602.1
Mouse 18S	5′ ACGAGACTCTGGCATGCTAACTAGT 3′	5′ CGCCACTTGTCCCTCTAAGAA 3′	7SYS_2

## Data Availability

RCSI has signed up to the FAIR data principles and details of this study will be available in the institutional online repository.
